# Theoretical Investigation of a Coumarin Fluorescent Probe for Distinguishing the Detection of Small-Molecule Biothiols

**DOI:** 10.3390/molecules29030554

**Published:** 2024-01-23

**Authors:** Yue Deng, He Huang, Jian Feng, Yongjin Peng, Yuling Liu

**Affiliations:** College of Modern Industry of Health Management, Jinzhou Medical University, Jinzhou 121001, China

**Keywords:** fluorescent probe, biothiols, theoretical investigation, electron excitation

## Abstract

Monitoring the level of biothiols in organisms would be beneficial for health inspections. Recently, 3-(2′-nitro vinyl)-4-phenylselenyl coumarin as a fluorescent probe for distinguishing the detection of the small-molecule biothiols cysteine/homocysteine (Cys/Hcy) and glutathione (GSH) was developed. By introducing 4-phenyselenium as the active site, the probe CouSeNO_2_/CouSNO_2_ was capable of detecting Cys/Hcy and GSH in dual fluorescence channels. Theoretical insights into the fluorescence sensing mechanism of the probe were provided in this work. The details of the electron excitation process in the probe and sensing products under optical excitation and the fluorescent character were analyzed using the quantum mechanical method. All these theoretical results would provide insight and pave the way for the molecular design of fluorescent probes for the detection of biothiols.

## 1. Introduction

Biothiols are involved in many processes of transfer and detoxification, including cell growth, redox, and so on. Small molecular biothiols, including cysteine, homocysteine, and glutathione (Cys, Hcy, and GSH, respectively), are important sulfur compounds that could protect parts of the body due to their reducibility [1,2,3]. Biothiols with structural differences would lead to different functions; meanwhile, the biothiols are related to each other. Cys is involved in the process of enzyme catalysis, detoxification, and protein synthesis. Hcy is a regulatory intermediate in the Met cycle and the precursors of Cys and methionine. GSH has a role in maintaining redox homeostasis in biological systems.

The concentration of biological biothiols will deviate from normal values under the influence of adverse factors and directly affect their functions. In this situation, diseases such as growth retardation, cardiovascular disease, liver damage, and rheumatism, etc., could be caused. Therefore, monitoring the level of biothiols in organisms would be beneficial for health inspections. Nowadays, the methods of detecting biothiols are diversified and gradually improved. Yet, different detection methods have their own advantages and drawbacks [2,4,5,6,7,8,9,10].

At present, the main methods for the detection and analysis of active sulfur species (RSS) include the high-performance liquid chromatography (HPLC) analytical method, the colorimetric method, mass spectrometry, electrochemical analysis, capillary electrophoresis, and fluorescence analysis [11,12,13,14,15,16]. 

According to the comparative analysis, the detection results of high-performance liquid chromatography and mass spectrometry are relatively stable and sensitive but necessitate complicated sample operations and expensive equipment. The capillary electrophoresis detection method is economical and rapid, but has slightly inferior sensitivity. Colorimetry is easy to use but usually produces a relatively big error. Although the electrochemical analysis method has the advantages of convenience and high sensitivity, it is relatively weak in terms of selectivity. In contrast, fluorescent probes have been successfully applied to many detection fields due to their advantages of high sensitivity, low background interference, high selectivity, and good biocompatibility. Combined with focusing microscope instruments, fluorescent probes are applied to real-time and in situ imaging of biological cells and tissues without causing any damage, which provides a powerful analytical technique for disease diagnosis and is becoming a popular detection method in biological and medical fields.

In recent years, remarkable progress has been made in the construction of biothiol fluorescent probes [4,17,18,19]. Many reported fluorescent probes can be responded to biothiols in cells, tissues, and a variety of amino acids. However, due to the similar structure and reactivity of biothiols, most of the fluorescent probes reported so far cannot distinguish between the biothiols of Cys, Hcy, and GSH, which hinders the research of their roles in corresponding physiological and pathological processes [20,21]. 

Recently, Chen et al. developed 3-(2′-nitro vinyl)-4-phenylselenyl coumarin as a fluorescent probe for distinguishing between the detection of Cys/Hcy and GSH. By introducing 4-phenyselenium as the active site, the probe CouSeNO_2_/CouSNO_2_ was capable of detecting Cys/Hcy and GSH in dual fluorescence channels [22]. For the biothiols, the first-step sensing reaction was experimentally proven to be the nucleophilic substitution of 4-phenylselenium with the thiol group. Furthermore, through two-channel fluorescent imaging, the probe CouSeNO_2_/CouSNO_2_ had been successfully applied to sense the exogenous and endogenous biothiols in living cells. Except for the Michael addition as a usual sensing reaction in reported nitroolefin fluorescent probes, the nucleophilic substitution of 4-phenylselenium in the probe CouSeNO_2_/CouSNO_2_ with the thiol group of a biothiol as the first-step sensing reaction not only accelerated the reaction to biothiols but also realized the distinction between Cys/Hcy and GSH in dual fluorescence channels. Compared with the experimental results, the theoretical research on the electronic structure, reaction sites, sensing mechanism, and fluorescent properties of the probe CouSeNO_2_/CouSNO_2_ in this work could provide insights and pave the way for the molecular design of fluorescent probes for the detection of biothiols.

## 2. Results and Discussion

**Figure 1:**
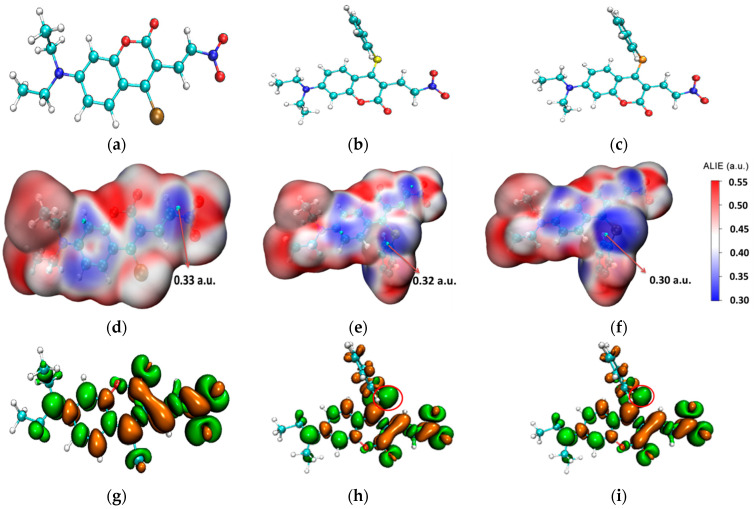
(**a**–**c**) The stable molecular structures of the CouClNO_2_, CouSNO_2_, and CouSeNO_2_ probes; (**d**–**f**) the surface map of ALIE on the CouClNO_2_, CouSNO_2_, and CouSeNO_2_ probes; and (**g**–**i**) the dual descriptors of the CouClNO_2_, CouSNO_2_, and CouSeNO_2_ probes. (the red circle indicate S and Se atom in the CouSNO_2_ and CouSeNO_2_ probes respectively).

The stable molecular structures of the probes CouClNO_2_, CouSNO_2_, and CouSeNO_2_ are shown in Figure 1a–c. Due to no apparent spectral response with biothiols, the probe CouClNO_2_ was only presented for structural comparison here but not for consideration in the following theoretical research.

From the surface map of average local ionization energy (ALIE) [23] on three probes in Figure 1d–f, it could be deduced that the C=C bond in CouClNO_2_ is the potential electrophilic reaction site (with an ALIE value of 0.33 a.u.); otherwise, the S(Se) atom and C=C bond in the CouSNO_2_ and CouSeNO_2_ probes are the potential electrophilic reaction sites (with the ALIE values of 0.32 a.u. and 0.30 a.u. for the S and Se atoms in the CouSNO_2_ and CouSeNO_2_ probes, respectively). 

Fukui function and dual descriptor, known as the important concepts in density functional reactivity theory, which was initially developed by Parr, are very popular methods for predicting reaction sites defined under the conceptual density functional theory framework [24,25,26]. The dual descriptors of the CouClNO_2_, CouSNO_2_, and CouSeNO_2_ probes were obtained through Multiwfn 3.8(dev) analysis based on the ORCA output results and are illustrated in Figure 1g–i. The S and Se atoms in the CouSNO_2_ and CouSeNO_2_ probes (indicated by the red circle in Figure 1h,i) were indicated to be the potential electrophilic reaction sites with biothiols, which were in agreement with the corresponding experimental results [22]. The lower ALIE value of the Se atom compared to the S atom indicated the higher sensitivity of the CouSeNO_2_ probe to biothiols than the CouSNO_2_ probe, which was also testified within the experiment work.

**Figure 2:**
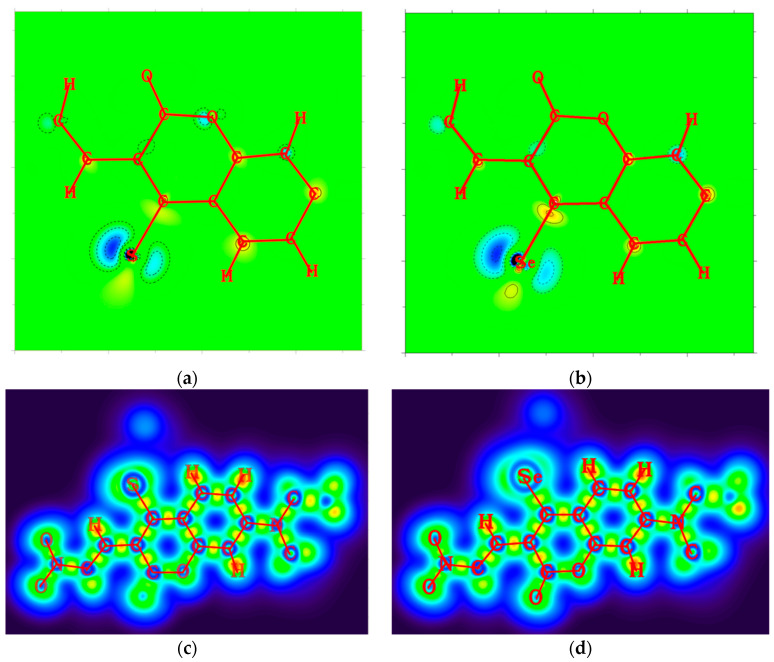
(**a**,**b**) Two-dimensional plots of dual descriptors on the molecular planes of the CouSNO_2_ and CouSeNO_2_ probes; (**c**,**d**) 2D lol on the molecular planes of the CouSNO_2_ and CouSeNO_2_ probes.

**Scheme 1:**
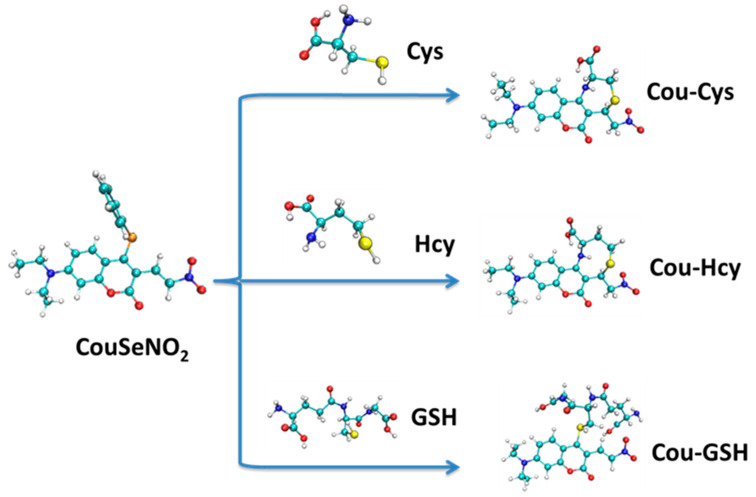
The sensing mechanism of CouSeNO_2_ towards biothiols.

From the 2D plots of dual descriptors on the main molecular planes of the CouSNO_2_ and CouSeNO_2_ probes, as shown in Figure 2a,b, the dual descriptor absolute values of the S and Se atoms are obviously larger than the values at other places within the probe molecule. This result indicated that a substitution reaction would likely occur within the S and Se atoms when the CouSNO_2_ and CouSNO_2_ probes encountered the biothiols. The 2D localized orbital locator (lol) on the molecular planes of the CouSNO_2_ and CouSeNO_2_ probes, as shown in Figure 2c,d, also indicated that the S and Se atoms in the probe molecules were potential reaction sites. The sensing mechanism of CouSeNO_2_ towards biothiols is shown in Scheme 1. 

**Figure 3:**
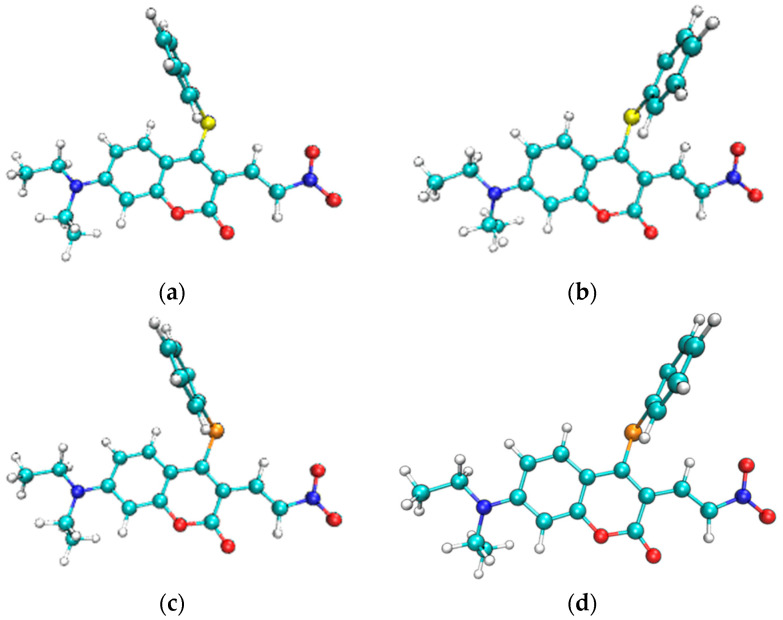
(**a**,**b**) Geometric structures of the S_0_ and S_1_ of the CouSNO_2_ probe; and (**c**,**d**) geometric structures of the S_0_ and S_1_ of the CouSeNO_2_ probe.

**Figure 4:**
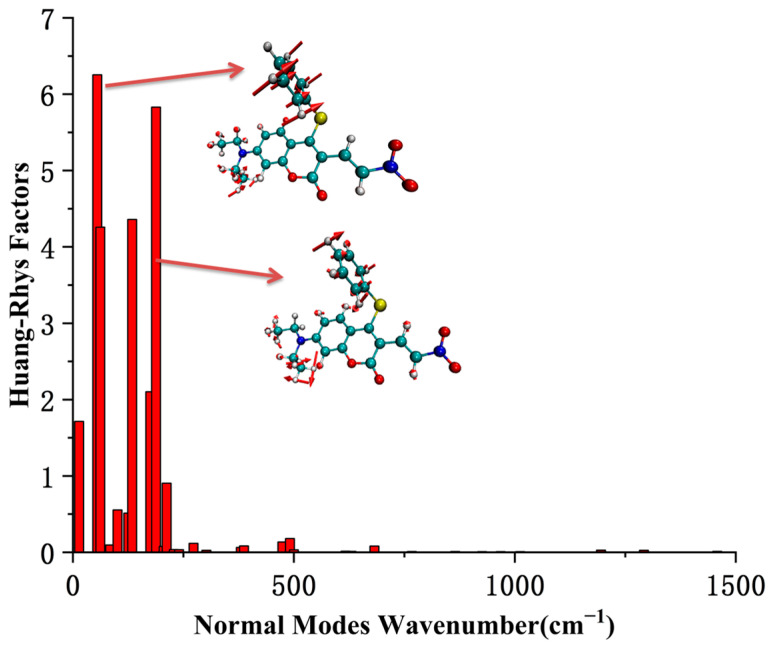
The Huang–Rhys factors of the CouSNO_2_ probe.

The most stable geometric structures of the ground state S_0_ and first excited state S_1_ of the CouSNO_2_ and CouSeNO_2_ probes are shown in Figure 3. It indicated a similar difference between the S_0_ and S_1_ structures of the CouSNO_2_ and CouSeNO_2_ probes, in which the benzene ring showed an obvious flip from the ground state to the first excited state. The dihedral angle, α, between the benzene ring and the main molecular plane of the CouSNO_2_ probe variated from 59° to 108° when the molecule was excited from S_0_ to S_1_; this change in α was from 56° to 108° in the CouSeNO_2_ probe. This large structural difference between S_0_ and S_1_ within the CouSNO_2_ and CouSeNO_2_ probes would lead to large reorganization energy and Huang–Rhys factors [27,28] for some normal vibration modes, as shown in Figure 4 (CouSNO_2_ was only shown for clarity consideration). It could be seen that the vibration mode with large Huang–Rhys factors were just corresponding with the swing of the benzene ring in the probe molecule. The reorganization energy and Huang–Rhys factors between S_0_ and S_1_ of the CouSNO_2_ and CouSeNO_2_ probes were calculated through the Dushin program [29].

**Figure 5:**
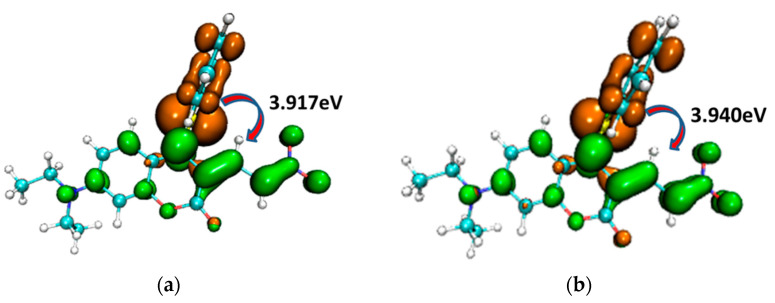
Hole–electron (brown and green colors, respectively) analysis for the electron excitation process from S_0_ to S_1_ within the (**a**) CouSNO_2_ and (**b**) CouSeNO_2_ probes.

To illustrate the electron excitation process from S_0_ to S_1_ within the CouSNO_2_ and CouSeNO_2_ probes, the hole–electron (brown and green colors, respectively, in Figure 5) analyses were performed based on the TDDFT results. It could be informed that the electron was mainly excited from the benzene ring part to the main planar part of the probes. The excitation energy from S_0_ to S_1_ in CouSeNO_2_ (3.940 eV) was a little larger than that in CouSNO_2_ (3.917 eV). 

**Figure 6:**
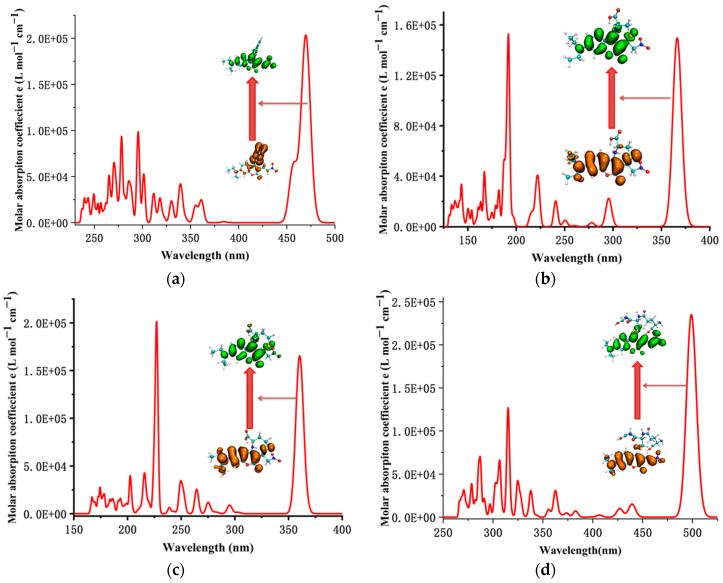
The simulated UV–Vis absorption spectrum of the probe and sensing products. (**a**) CouSeNO_2_. (**b**) Cou-Cys. (**c**) Cou-Hcy. (**d**) Cou-GSH.

The simulated UV–Vis absorption spectrum of the CouSeNO_2_ probe, as shown in Figure 6a, indicated that the absorption wavelength from S_0_ to S_1_ was about 473 nm, which was near the experimental value of 480 nm and testified to the reasonable choice of the functional and basis set for electron excitation calculation on this kind of organic molecular probe. After the reaction with Cys and Hcy, the absorption wavelengths from S_0_ to S_1_ of the sensing products Cou-Cys and Cou-Hcy were changed by a blue shift to be about 360 nm and 357 nm, respectively, which were consistent with the experimental results. 

Unlike the charge transfer characteristic of electron excitation in the process from S_0_ to S_1_ within the original CouSeNO_2_ probe, it was shown the local excitation character for the electron excitation process from S_0_ to S_1_ within the sensing products Cou-Cys and Cou-Hcy, and this local excitation character led to a significant increase in the fluorescent intensity at about 460 nm and 451 nm, respectively, which were testified within both the theoretical and experimental results. A similar reaction between the CouSeNO_2_ probe and GSH occurred, which also led to the variation in the UV–Vis absorption spectrum and fluorescent intensity of the sensing product Cou-GSH. Without the seven- or eight-membered ring like in sensing products Cou-Cys and Cou-Hcy, due to the Michael addition reaction of the thiol group to the unsaturated C=C double bond, there was a red shift within the UV–Vis absorption and fluorescent spectrum of sensing product Cou-GSH compared with the original probe CouSeNO_2_. The theoretical absorption and emission wavelength between S_0_ and S_1_ was about 500 nm and 550 nm, respectively, which were well agreed with the experimental values of 515 nm and 562 nm, respectively. The theoretical and experimental fluorescent-related absorption and emission wavelengths are summarized in Table 1 and Table 2.

**Figure 7:**
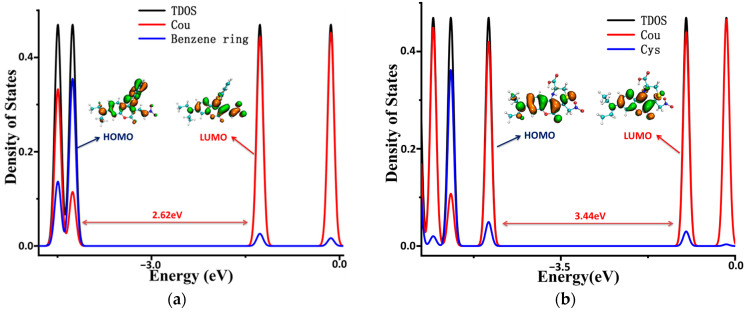

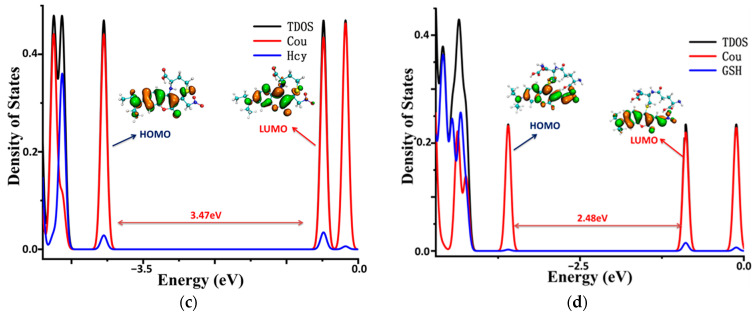
The density of the electronic states of the probe and sensing products. (**a**) CouSeNO2. (**b**) Cou-Cys. (**c**) Cou-Hcy. (**d**) Cou-GSH.

To illustrate the electronic structures of the CouSeNO_2_ probe and its sensing product with biothoils in more depth, the density of electronic states (DOSs) were calculated and are illustrated in Figure 7. The main orbital transition contribution to the electron excitation between S_0_ and S_1_ in the probes and sensing products was the highest occupied molecular orbit (HOMO) and the lowest unoccupied molecular orbit (LUMO), as shown in Table 1 and Table 2. The fluorescence of the probes and sensing products were decided through the electron radiation process from S_1_ to S_0_. 

The total DOS (TDOS) of the probe and sensing product molecules and the partial DOS of two individual parts in the molecules (coumarin part and benzene ring in the probe and biothoils in the sensing product) are all depicted within Figure 7. It could be seen that the obvious charge transfer characteristic in the electron excitation process between S_0_ and S_1_ in the CouSeNO_2_ probe, in which the HOMO was mainly contributed by the benzene ring part and the LUMO was mainly contributed by the coumarin part. This charge transfer character indicated that the ICT process led to the small oscillation strength between the S_0_ and S_1_ states and a weak fluorescent intensity in the original CouSeNO_2_ probe. Otherwise, the local excitation characteristic was shown in the electron excitation process between S_0_ and S_1_ in the sensing products through the probe reaction with the biothiols, which led to the corresponding significant oscillation strength and fluorescent intensity. Due to the different molecular structures of the biothoils, the sensing product Cou-GSH without its 7–8-membered rings showed a wavelength red shift in maximum absorption peak and fluorescence compared with the original CouSeNO_2_ probe. Contrarily, the Michael addition reaction between the thiol groups (Cys and Hcy) and the unsaturated C=C double bond in the CouSeNO_2_ probe led to the formation of the 7–8-membered rings in the sensing products Cou-Cys and Cou-Hcy, which made the different electronic structure variation compared with the original probe CouSeNO_2_. Both the wavelength of the maximum absorption peak and fluorescence took a blue shift relative to the CouSeNO_2_ probe. The blue and red absorption shifts of CouSeNO_2_ with Cys, Hcy, and GSH were clearly related to the HOMO/LUMO energy gaps of the corresponding sensing products. So, the different wavelengths and colors of the fluorescence from the sensing product with the biothiols (Cys, Hcy, and GSH) allowed for the CouSeNO_2_ probe to be successfully applied in distinguishing the detection of the small-molecule biothiols.

## 3. Theoretical Methods

The theoretical methods of the research for the fluorescent probe CouSeNO_2_/CouSNO_2_ sensing biothiols were as follows:

1. The functional and basis set combination CAM-B3LYP/def2-TZVPD was used in structure optimization, corresponding vibrational frequency analysis on the probe, and sensing product conformations with ORCA program 5.1 [30,31,32,33]. Non-imaginary frequency was found in the vibrational analysis on the stable geometric structure, which confirmed the stability of the structure optimization results. The wB2GP-PLYP/def2-TZVPD combination was used in single-point energy to obtain free energy with high precision, according to benchmark research [34]. Similar calculated results were obtained in the gas phase and in several solvents with different polarities, which indicated that this fluorescent probe was insensitive to the solvent effect.
2. The electronic structure and fluorescent properties of the probe and its sensing products were obtained through the Multiwfn 3.8(dev) code [35] based on the DFT and TDDFT results through the ORCA program.
3. The reorganization energy and Huang–Rhys factors between the S_0_ and S_1_ states of the probe and sensing products were obtained through the Dushin program.
4. Most of the figures in this work were rendered by means of VMD 1.9.3 software [36].

## 4. Conclusions

The electron structure and fluorescent theoretical analysis indicated a local excitation character for the electron excitation process from S_0_ to S_1_ within the sensing product of the CouSeNO_2_ probe’s reaction with small-molecule biothiols, including Cys/Hcy and GSH. Due to the different molecular structures of the biothoils, the sensing product Cou-GSH without its 7–8-membered rings showed a wavelength red shift in its maximum absorption peak and fluorescence compared with the original CouSeNO_2_ probe. Contrarily, the Michael addition reaction between the thiol groups (Cys and Hcy) and the unsaturated C=C double bond in the CouSeNO_2_ probe led to both the wavelengths of the maximum absorption peak and fluorescence taking a blue shift relative to the CouSeNO_2_ probe. So, the different wavelengths and colors of the fluorescence from the sensing product with the biothiols (Cys, Hcy, and GSH) allowed for the CouSeNO_2_ probe to be successfully applied in distinguishing the detection of the exogenous and endogenous biothiols in living cells. The theoretical investigation of the mechanism of fluorescent probe molecular design would provide insights into building highly efficient fluorescent probes for biothiol detection in the future.

## Figures and Tables

**Table 1:** The main electron excitation processes in the probe and sensing product molecule.

Probe/Sensing Product	Electronic Transition ^a^	Excitation Energy Theoretical/Experimental (nm)	Oscillator Strength	Composition ^b^	CI ^c^
CouSeNO_2_	S_0_ → S_1_	473/480	0.1063	H → L	0.6815
Cou-Cys	S_0_ → S_1_	360/370	0.7921	H → L	0.6908
Cou-Hcy	S_0_ → S_1_	357/365	0.8014	H → L	0.7014
Cou-GSH	S_0_ → S_1_	500/515	0.7318	H → L	0.7219

^a^ Only the excited states with an oscillator strength larger than 0.1 were considered. ^b^ H stands for HOMO and L stands for LUMO. ^c^ Coefficient of the wave function for each excitation was in absolute value.

**Table 2:** The main electron emission processes in the probe and sensing product molecule.

Probe/Sensing Product	Electronic Transition ^a^	Emission Energy Theoretical/Experimental (nm)	Oscillator Strength	Composition ^b^	CI ^c^
CouSeNO_2_	S_1_ → S_0_	498/N.A.	0.0245	H → L	0.6726
Cou-Cys	S_1_ → S_0_	460/465	0.7146	H → L	0.7017
Cou-Hcy	S_1_ → S_0_	451/457	0.7219	H → L	0.7102
Cou-GSH	S_1_ → S_0_	550/562	0.6849	H → L	0.7115

^a,b,c^ same indication as in Table 1.

## Data Availability

The data presented in this study are available in article.
